# Human Microcephaly Protein *RTTN* Is Required for Proper Mitotic Progression and Correct Spindle Position

**DOI:** 10.3390/cells10061441

**Published:** 2021-06-09

**Authors:** En-Ju Chou, Tang K. Tang

**Affiliations:** Institute of Biomedical Sciences, Academia Sinica, Taipei 11529, Taiwan; enjuchou0531@gmail.com

**Keywords:** primary microcephaly, *MCPH*, centriole, centrosome, cell division, neural progenitors

## Abstract

Autosomal recessive primary microcephaly (*MCPH*) is a complex neurodevelopmental disorder characterized by a small brain size with mild to moderate intellectual disability. We previously demonstrated that human microcephaly *RTTN* played an important role in regulating centriole duplication during interphase, but the role of *RTTN* in mitosis is not fully understood. Here, we show that *RTTN* is required for normal mitotic progression and correct spindle position. The depletion of *RTTN* induces the dispersion of the pericentriolar protein γ-tubulin and multiple mitotic abnormalities, including monopolar, abnormal bipolar, and multipolar spindles. Importantly, the loss of *RTTN* altered NuMA/p150Glued congression to the spindle poles, perturbed NuMA cortical localization, and reduced the number and the length of astral microtubules. Together, our results provide a new insight into how *RTTN* functions in mitosis.

## 1. Introduction

In most animal cells, the centrosome is the major microtubule-organizing center (MTOC). It is composed of a pair of centrioles surrounded by pericentriolar material (PCM). Centrioles are barrel-shaped structures composed of microtubules (MTs), and as basal bodies, they serve as a template for the formation of cilia and flagella [1,2,3]. Autosomal recessive primary microcephaly (*MCPH*) is a rare genetically heterogeneous disorder characterized by small brain size with mild to severe mental retardation [4]. A number of genes that encode centriole/centrosome proteins have been identified as causal genes of *MCPH*, including *MCPH1*, *WDR62*, *CDK5RAP2*, *CEP152*, *ASPM*, *CPAP/CENPJ*, *CEP63*, and *STIL* [5]. Interestingly, more than half of microcephaly proteins were found to cooperate with each other and participate in centriole biogenesis and/or centrosome function. 

We and others previously found that human microcephaly protein *CPAP* (centrosomal protein 4.1-associated protein) plays a key role in the regulation of centriole duplication, particularly in the control of centriole length [6,7,8]. We further demonstrated that various human microcephaly proteins could interact with each other, including *CPAP*-*STIL* [9], *CPAP*-SAS6-*CEP135* [10], and *RTTN*-*STIL* [11], which are required for building a daughter centriole. In addition, it was reported that human microcephaly protein *CEP152* interacts with not only *CEP63* [12] but also *CPAP* [13], and *WDR62* also forms a complex with *ASPM* and *CEP63* that regulates centriole biogenesis [14]. These findings led us to propose that a defect in centriole biogenesis in neural progenitors is one of the key pathways leading to *MCPH* [5,9,10,11].

*RTTN* (also known as Rotatin) is an evolutionarily conserved centrosome-associated protein found in many organisms. The *RTTN* gene was initially identified in a homozygous mutant mouse that shows defects in axial rotation, left–right specification, and embryonic lethality [15], and Ana 3 (a fly homologue of *RTTN*) was previously reported to be required for structural integrity of centrioles and basal bodies [16]. Interestingly, we found that complete loss of *RTTN* impairs centriole biogenesis and induces primitive procentriole bodies (PPBs) that contain the early-born centriolar proteins (e.g., SAS-6, *STIL*, and *CPAP*) but lack the later-born proteins (POC5 and POC1B), suggesting that the later process of centriole assembly is inhibited during the S phase in interphase cells [11]. 

Recently, a number of *RTTN* gene mutations have been identified in human patients with primary microcephaly associated with primordial dwarfism [17,18], lissencephaly associated with simplified gyral pattern [19,20], polymicrogyria associated with seizures [21], and pontocerebellar hypoplasia [20]. Interestingly, skin fibroblasts from patients with *RTTN* gene mutations exhibit mitotic defects, leading to aneuploidy and apoptosis [22]. However, the fundamental roles of *RTTN* in mitosis are not completely understood. Here, we reported that loss of *RTTN* disrupted normal cell progression, including G2/M phase arrest and increased aneuploidy cells. Additionally, *RTTN* deletion results in PCM dispersion and multiple mitotic abnormalities, including monopolar, abnormal bipolar, or multipolar spindles. Moreover, loss of *RTTN* caused the spindle positioning defects, such as mis-localization of cortical NuMA and reduction of the number and the length of astral microtubules. A possible mechanism of how *RTTN* participates in mitosis is discussed.

## 2. Materials and Methods

### 2.1. Cell Culture and Reagents

Human telomerase-immortalized retinal pigment epithelial cells (hTERT-RPE1) and hTERT-RPE1-derived cell lines, such as *RTTN^+/+^*; *p53^+/+^*, *RTTN^+/+^*; *p53^−/−^*, and *RTTN^−/−^*; *p53^−/−^*-based *RTTN*-wild-type-GFP-inducible cells (*RTTN*-WT-GFP) [11] were maintained in DME/F12 (1:1) medium supplemented with 10% fetal bovine serum (FBS). To synchronize cells at G2/M phase, cells were treated with 2 μg/mL aphidicolin (Sigma-Aldrich, St. Louis, MO, USA) for 24 h and then released in fresh medium for 17 h. To further enrich the metaphase cells, the treated cells were incubated with 5 μM MG132 (Sigma-Aldrich) for 1 h before fixation. In nocodazole experiments, cells were treated with 20 nM of nocodazole (Sigma-Aldrich) for 1 h in the medium containing 5 μM MG132.

### 2.2. Antibodies

The antibodies against *RTTN* (1:500 dilution; rabbit polyclonal raised against *RTTN*, residues 1347-1591) [11] and CEP120 (1:1000 dilution; rabbit polyclonal raised against CEP120, residues 639-986) [23] were obtained as described previously. Other commercially available antibodies used in this study included anti-hPOC5 (Bethyl Laboratories, Inc., Montgomery, TX, USA; 1:500 dilution), anti-α-tubulin (DM1α, Sigma-Aldrich; 1:300 dilution), anti-γ-tubulin (GTU88, Sigma-Aldrich; 1:300 dilution), anti-CDK5RAP2 (Bethyl Laboratories, Inc.; 1:1000 dilution), anti-NuMA (Abcam; 1:500 dilution), anti-p150Glued (Transduction Laboratories; 1:300 dilution), and anti-pericentrin (BD Transduction Laboratories, Franklin Lakes, NJ, USA; 1:1000 dilution).

### 2.3. Immunofluorescence Confocal Microscopy

Cells grown on coverslips were fixed in methanol at −20 °C for 5 min. To examine the astral microtubules, cells were pre-extracted with 0.5% Triton X-100 in PHEM buffer (120 mM PIPES, 50 mM HEPES, 20 mM EGTA, 8 mM MgSO_4_) for 1 min at 37 °C then fixed in methanol at −20 °C for 5 min. The fixed cells were then blocked with 10% normal goat serum in PBS and incubated with the indicated primary antibodies. After being washed with PBST (PBS containing 0.1% Tween-20), the cells were incubated with Alexa Fluor 488-, Alexa Fluor 568-, or Alexa Fluor 647-conjugated secondary antibodies (Invitrogen). DNA was counterstained with DAPI (4,6-diamidino-2-phenylindole). The samples were mounted in Vectashield mounting media (Vector Laboratories) and visualized on a confocal microscope (LSM 880; Carl Zeiss, Jena, Germany) with a Plan Apochromat 63x/1.4 NA oil-immersion objective. Images were acquired with the ZEN software (Carl Zeiss).

### 2.4. Immunoblotting

To examine the protein level of NuMA, p150Glued, and *RTTN*, cells were lysed in RIPA buffer (50 mM Tris-HCl, pH 8.0, 150 mM NaCl, 1% NP-40, 0.5% sodium deoxycholate, 20 mM β-glycerophosphate, 20 mM NaF, 1 mM Na3VO4, and protease inhibitors including 1 mg/mL leupeptin, 1 mg/mL pepstatin, and 1 mg/mL aprotinin) for 30 min at 4 °C. The cell lysates were centrifuged at 16,000× *g* at 4 °C for 15 min, separated by SDS-PAGE, and probed with appropriate antibodies. 

### 2.5. Flow Cytometry Analysis

To analyze the cell cycle, cells were fixed in ice-cold 100% ethanol for 30 min, washed with PBS, and stained with 40 µg/mL propidium iodide at room temperature for 30 min. Cells were then analyzed by flow cytometer (Attune-NxT; Thermo Fisher Scientific, Waltham, MA, USA).

### 2.6. Spindle Orientation Analysis

To examine whether *RTTN* depletion may cause the spindle orientation defect, we measured the linear distance of the xy plane and the vertical distance of the z plane between the two spindle poles (the maximum intensity of CDK5RAP2 signal was used as the spindle pole marker) of metaphase cells and calculated the spindle angles α. Z-stack images (0.95 μm per stack) of metaphase cells stained with antibodies against CDK5RAP2 and α-tubulin were analyzed using the ZEN software (Carl Zeiss).

### 2.7. Statistics

To determine the significance among the two or more experimental conditions, an unpaired two-tailed Student’s t test or one-way ANOVA with Tukey’s multiple comparison test (GraphPad Prism 5 software) was used for analysis. Data are presented as the mean ± standard error of the mean (SEM). * *p* < 0.05, ** *p* < 0.01, and *** *p* < 0.0001 were considered statistically significant.

## 3. Results

### 3.1. RTTN Is Required for Proper Mitotic Progression and Maintenance of Spindle Pole Integrity

**Figure 1:**
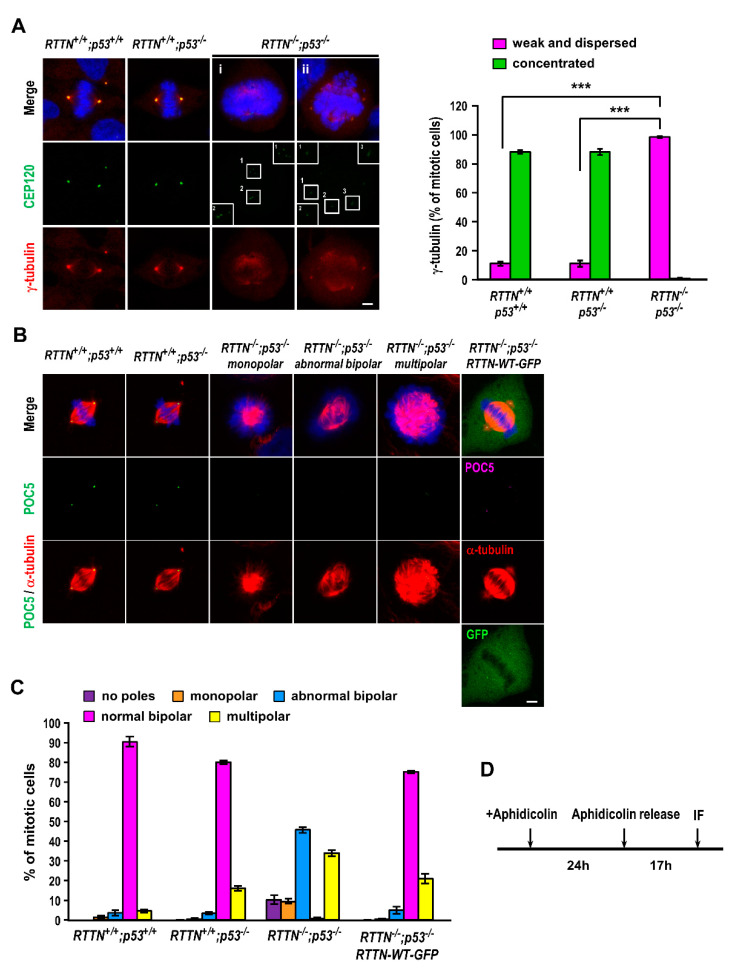
*RTTN* depletion resulted in multiple mitotic abnormalities. RPE1-based *RTTN^+/+^*; *p53^+/+^*, *RTTN^+/+^*; *p53^−/−^*, or *RTTN^−/−^*; *p53^−/−^* mitotic cells were immuno-stained with antibodies against CEP120 and γ-tubulin ((**A**), the quantitation of γ-tubulin distribution pattern is shown in the right panel), or POC5 and α-tubulin (**B**). DNA was counterstained with DAPI. In (**B**), the mitotic abnormalities in a portion of *RTTN^−/−^*; *p53^−/−^* cells can be effectively rescued to normal bipolar spindles by exogenously expressing the *RTTN*-WT-GFP construct. (**C**) Histograms illustrating the percentages of mitotic cells with indicated mitotic phenotypes. (**D**) Schematic of the procedure used to enrich mitotic cells. Data are represented as the mean ± SEM from four independent experiments. Scale bars: 5 μm. *** *p* < 0.0001.

To investigate the role of *RTTN* in mitosis, we took advantage of previously established *RTTN* gene knockout (KO) in hTERT-RPE1-based p53-deficient cells (*p53*^−/−^; *RTTN*^−/−^) to investigate the role of *RTTN* in mitosis, as homozygous *RTTN* deletion is lethal to the cells containing wild-type p53 [11]. We first examined the PCM protein γ-tubulin, a known microtubule nucleating factor located at the spindle poles, in synchronized *RTTN*^−/−^; *p*53^−/−^ cells during mitosis. Our results show that almost all the *RTTN*^−/−^; *p*53^−/−^ cells (99% ± 1%) exhibited weak and dispersed γ-tubulin (Figure 1A, the quantitation is shown in the right panel) compared to control cells (*RTTN^+/+^*; *p53^+/+^* and *RTTN^+/+^*; *p53^−/−^* cells), in which γ-tubulin was concentrated and focused on the spindle poles, suggesting that *RTTN* is required for spindle pole integrity in mitosis. We previously showed that complete loss of *RTTN* in p53-deficient cells (*RTTN*^−/−^; *p*53^−/−^) induces amplification of primitive procentriole bodies (PPBs) that contained early born centriolar proteins (e.g., SAS-6, *STIL*, *CPAP*, and CEP120) but lacked later-born proteins (POC5, POC1B) in interphase cells [11]. Here, we found that the CEP120-containing PPBs appear to be associated with abnormal spindle poles with diffuse γ-tubulin signals in *RTTN*^−/−^; *p*53^−/−^ mitotic cells (Figure 1A(i–ii)). We next examined the effects of mitotic phenotypes in *RTTN* knockout cells by immunostaining of α-tubulin and POC5 (a later-born centriolar protein). As shown in Figure 1B, multiple mitotic abnormalities were observed in *RTTN*^−/−^; *p*53^−/−^ cells, including no poles (very rare), monopolar spindle, abnormal bipolar spindles (asymmetric bipolar or unfocused spindle poles), and multipolar spindles. The percentages of abnormal bipolar and multipolar spindles were significantly increased in *RTTN*^−/−^; *p*53^−/−^ cells (abnormal bipolar, 46 ± 1%; multipolar, 34 ± 2%) compared to *RTTN^+/+^*; *p53^+/+^* and *RTTN^+/+^*; *p53^−/−^* control cells (Figure 1B,C). Importantly, the multiple mitotic abnormalities can be effectively rescued and restored to normal bipolar spindles by exogenously expressing *RTTN*-WT-GFP, indicating that the abnormal mitotic phenotypes are specific to the loss of *RTTN* expression (Figure 1B,C).

**Figure 2:**
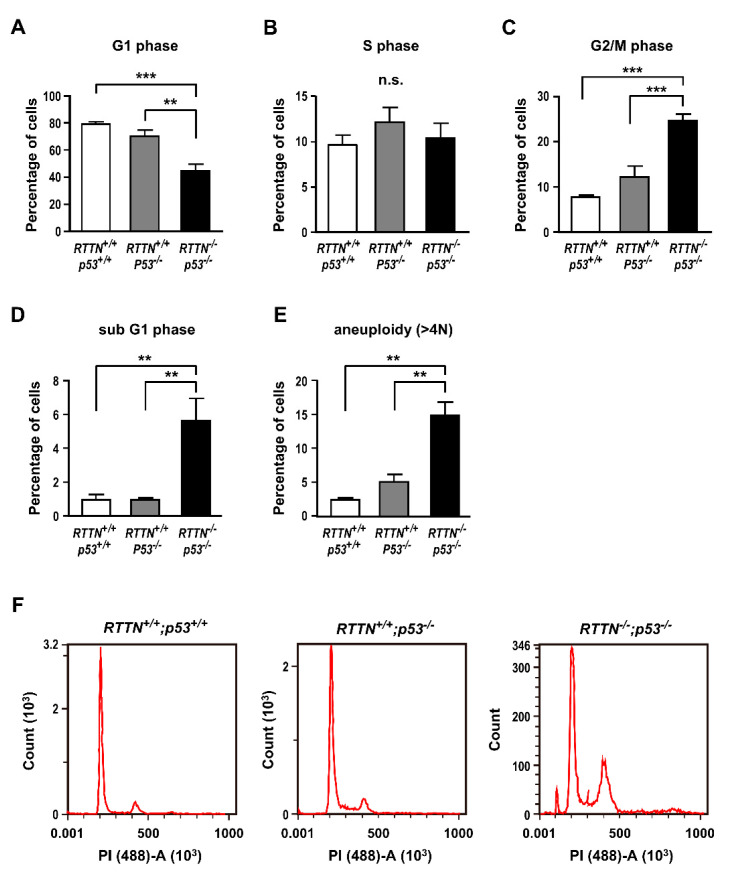
*RTTN* is required for proper mitotic progression. Flow cytometric analysis of cell cycle in *RTTN^+/+^*; *p53^+/+^*, *RTTN^+/+^*; *p53^−/−^*, or *RTTN^−/−^*; *p53^−/−^* cells after PI staining to measure the percentages of cells in G1 phase (**A**), S phase (**B**), G2/M phase (**C**), sub-G1 phase (**D**), and the cells with aneuploidy (>4 N) (**E**). (**F**) A representative chart of flow cytometric analysis. Data were analyzed by one-way ANOVA with Tukey’s multiple comparison test. Data are represented as the mean ± SEM from six independent experiments. *** *p* < 0.0001; ** *p* < 0.01. n.s. not significant.

We further analyzed whether the cell cycle progression was perturbed upon *RTTN* loss by flow cytometry. Our results showed that complete loss of *RTTN* reduced G1-phase cells (Figure 2A), with no change in S phase cells (Figure 2B) but increased G2/M-phase cells (Figure 2C). Furthermore, aneuploidy cells (>4 N) and apoptotic cells (sub-G1) were significantly increased in *RTTN*^−/−^; *p*53^−/−^ cells (14.9% ± 2.0% for aneuploidy; 5.6% ± 1.3% for apoptosis) compared to the control cells (*RTTN^+/+^*; *p53^+/+^* or *RTTN^+/+^*; *p53^−/−^*) (Figure 2D–F). Together, our results showed that *RTTN* is required for normal mitotic progression and spindle pole integrity.

### 3.2. Loss of RTTN Altered NuMA/p150Glued Distribution during Mitosis

**Figure 3:**
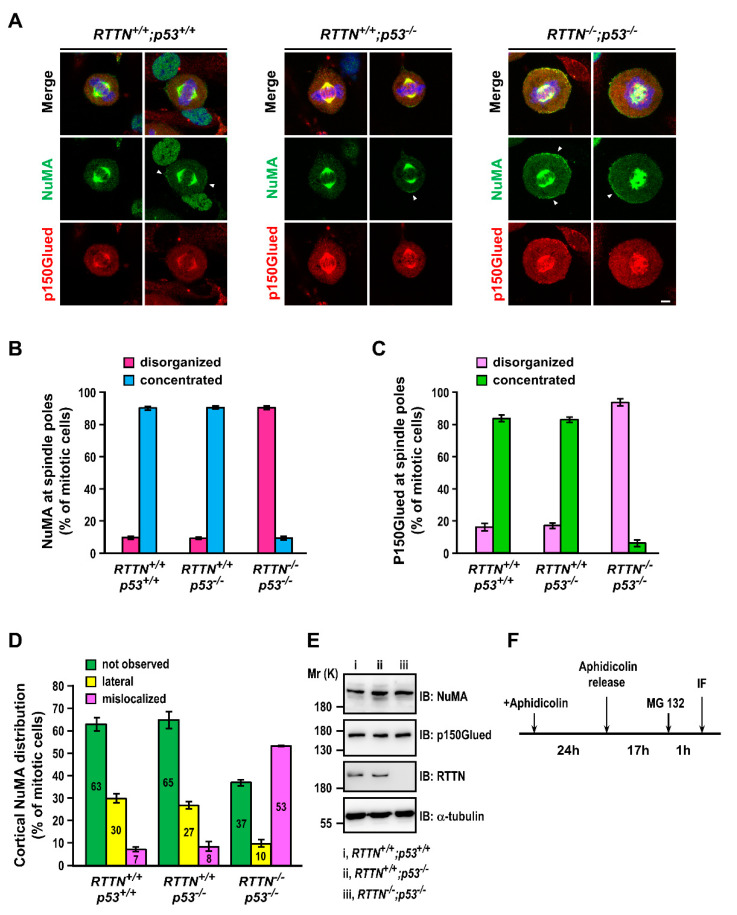
Loss of *RTTN* altered NuMA/p150Glued congression to the spindle poles and perturbs NuMA cortical localization during mitosis. RPE1-based *RTTN^+/+^*; *p53^+/+^*, *RTTN^+/+^*; *p53^−/−^*, or *RTTN^−/−^*; *p53^−/−^* mitotic cells were co-immuno-stained with anti-NuMA and anti-p150Glued antibodies, followed with confocal fluorescence microscopy (**A**). Arrowheads in (**A**) indicate the cortical NuMA. Histograms illustrating the percentages of mitotic cells with disorganized or concentrated NuMA (**B**) or p150Glued (**C**) at the spindle poles. Histograms in (**D**) illustrating the percentages of mitotic cells with various patterns of cortical NuMA distribution. We define the lateral distribution of NuMA as that NuMA localized at the cortical membrane, which is positioned laterally to the division axis. (**E**) RPE1-based *RTTN^+/+^*; *p53^+/+^*, *RTTN^+/+^*; *p53^−/−^*, or *RTTN^−/−^*; *p53^−/−^* cell lysates were analyzed by Western blotting using the indicated antibodies. A representative image of each blot is shown in (E). The uncropped images of Western blots are shown in Supplementary Figure S1. (**F**) Schematic of the procedure used to enrich mitotic cells. Data are represented as the mean ± SEM from three independent experiments. Scale bars: 5 μm.

It was reported that, during early mitosis, the interaction of NuMA with dynein and Eg5 is required to tether the microtubules to the pole and focus the astral microtubules to the centrosome [24,25,26]. To investigate whether loss of *RTTN* altered the localization of NuMA and p150Glued (a subunit of dynactin complex that acts as a co-factor for the microtubule motor cytoplasmic dynein) during mitosis, we performed immunofluorescence staining on cells with antibodies against NuMA and p150Glued. Our results showed that NuMA and p150Glued signals normally exhibited an umbrella shape that congressed to the spindle poles in the control cells (*RTTN^+/+^*; *p53^+/+^* and *RTTN^+/+^*; *p53^−/−^*) during mitosis (Figure 3A, left and middle panels), and their quantitation at the spindle poles is shown in Figure 3B (NuMA) and Figure 3C (p150Glued). 

In addition to the umbrella pattern, a portion of NuMA (~30%, Figure 3D) displayed a cortical localization laterally to the spindle poles at one end or both ends in control RPE1 metaphase cells (arrowheads in *RTTN^+/+^*; *p53^+/+^* and *RTTN^+/+^*; *p53^−/−^* cells, Figure 3A, left and middle panels). Interestingly, *RTTN* knockout caused the disruption of the umbrella-shaped spindle pole to become more disorganized in *RTTN*^−/−^; *p*53^−/−^ cells (Figure 3A, right panel; Figure 3B,C) but without protein loss of NuMA and p150Glued (Figure 3E). Intriguingly, the proportion of mis-localized cortical NuMA (from lateral distribution to random distribution) was significantly increased (~53%) in *RTTN*^−/−^; *p*53^−/−^ cells compared to the control groups (arrowheads in Figure 3A, quantitation in Figure 3D). Together, our results suggest that *RTTN* plays an essential role in regulating the spindle orientation and positioning, in which the NuMA/Dynein complex is involved.

### 3.3. RTTN Regulates Cortical NuMA Release Through Astral Microtubules and Loss of RTTN Causes Spindle Misorientation

**Figure 4:**
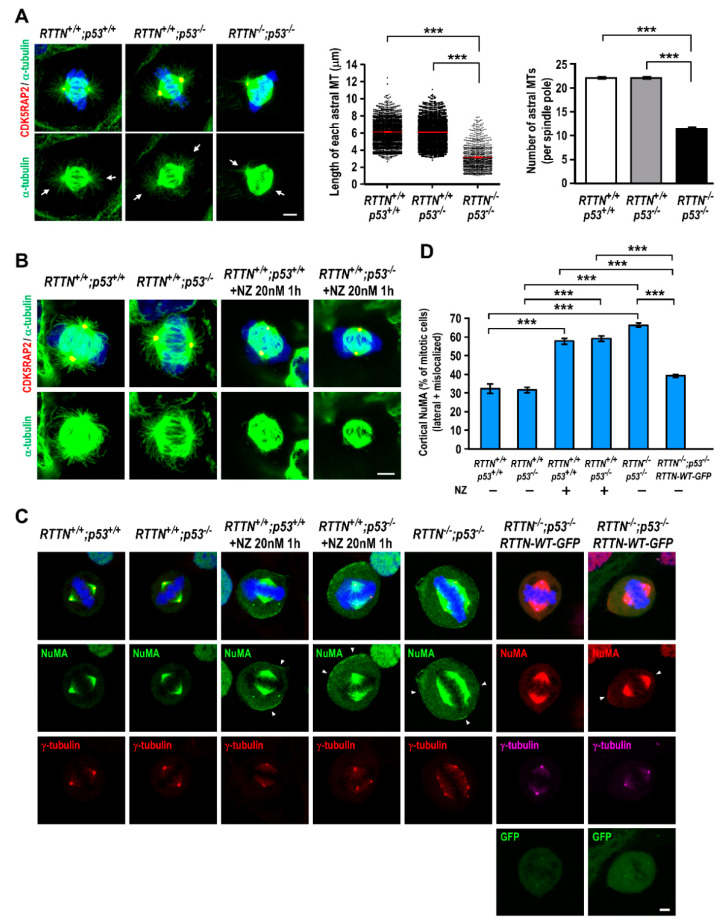
*RTTN* regulates cortical NuMA release through astral microtubules. RPE1-based *RTTN^+/+^*; *p53^+/+^*, *RTTN^+/+^*; *p53^−/−^*, or *RTTN^−/−^*; *p53^−/−^* cells were treated as described in Figure 3F. (**A**) The effects on astral microtubules after *RTTN* knockout. To easily visualize the astral microtubules, the cells were pre-extracted with PHEM, fixed in methanol, and immuno-stained with the indicated antibodies. Arrows in (**A**) indicate the astral microtubules. (**A**, middle panel) Quantification data of the length of each astral microtubule (*n* = 1989 for *RTTN^+/+^*; *p53^+/+^* group; *n* = 1988 for *RTTN^+/+^*; *p53^−/−^* group; *n* = 1032 for *RTTN^−/−^*; *p53^−/−^* group). (**A**, right panel) Histograms illustrating the number of astral microtubules per spindle pole (*n* = 90 for each group). Data are represented as the mean ± SEM from three independent experiments. (**B**,**C**) The effects of nocodazole (NZ) treatment in astral microtubule assembly (**B**) and cortical NuMA distribution (**C**). For nocodazole (NZ) treatment, cells were treated with or without 20 nM of NZ for 1 h in the presence of 5 μM MG132. After treatment, cells were immuno-stained with indicated antibodies, followed by confocal fluorescence microscopy. DNA was counterstained with DAPI. Arrowheads in (**C**) indicate the cortical NuMA. In the rescue experiment (**C**), the spindle pole integrity and the abnormal distribution and the retention of cortical NuMA could be partially rescued by exogenously expressing *RTTN*-WT-GFP in the *RTTN^−/−^; p53^−/−^* cells. (**D**) Histograms illustrating the percentages of metaphase cells with cortical NuMA (both lateral and mis-localized patterns). Data are represented as the mean ± SEM from three independent experiments. Data are analyzed by one-way ANOVA with Tukey’s multiple comparison test. *** *p* < 0.0001. Scale bars: 5 μm.

Spindle orientation and positioning was reported to be conducted by the interaction between astral microtubules and cortical proteins [27,28], and we found that cortical localization of NuMA was affected by *RTTN* knockout (Figure 3). We then examined the effect of astral microtubules upon *RTTN* knockout in metaphase cells. To visualize astral microtubules clearly, the cells were pre-extracted with PHEM buffer [29] to stabilize the microtubules, followed by immunostaining with antibodies against α-tubulin and CDK5RAP2 (a centrosome marker). Interestingly, the astral microtubule arrays were greatly affected in *RTTN^−/−^*; *p53^−/−^* cells (Figure 4A), since both the length (Figure 4A, middle panel) and the number (Figure 4A, right panel) of astral microtubules were significantly decreased, compared to that of the control cells (*RTTN^+/+^*; *p53^+/+^* and *RTTN^+/+^*; *p53^−/−^*, Figure 4A). Notably, the cortical NuMA (both lateral and mis-localized NuMA) were largely increased in *RTTN^−/−^*; *p53^−/−^* cells (~63%) compared to the control cells (~37% in *RTTN^+/+^*; *p53^+/+^* and ~35% in *RTTN^+/+^*; *p53^−/−^*) (Figure 3D).

Recently, it was reported that astral microtubules may regulate the release and transportation of cortical NuMA from cell cortex to spindle poles [30,31]. Consistent with previous findings [30], low-dose (20 nM) nocodazole (NZ) treatment that selectively disrupts the astral microtubules but preserves spindle microtubules (Figure 4B) could enhance cortical NuMA (lateral + mis-localized NuMA) at the cell cortex in the control mitotic cells (~58%, *RTTN^+/+^*; *p53^+/+^* and ~60%, *RTTN^+/+^*; *p53^−/−^*) (Figure 4C, D). Intriguingly, in the absence of NZ, the retention of cortical NuMA (lateral and mis-localized NuMA) at the cell cortex was greatly increased in *RTTN^−/−^*; *p53^−/−^* cells (~67%, Figure 4C, D), a pattern similar to low-dose NZ-treated control cells (~59%, Figure 4D). Importantly, this abnormal retention effect could be rescued by exogenously expressing *RTTN*-WT-GFP in *RTTN^−/−^*; *p53^−/−^* cells (Figure 4C,D). Furthermore, *RTTN*-WT-GFP could also partially rescue the mis-localized cortical NuMA to the right position (Figure 4C, the right-most panel). Collectively, our findings support the concept that loss of *RTTN* perturbs the assembly of astral microtubules, which consequently blocks the release of cortical NuMA from the cell cortex.

**Figure 5:**
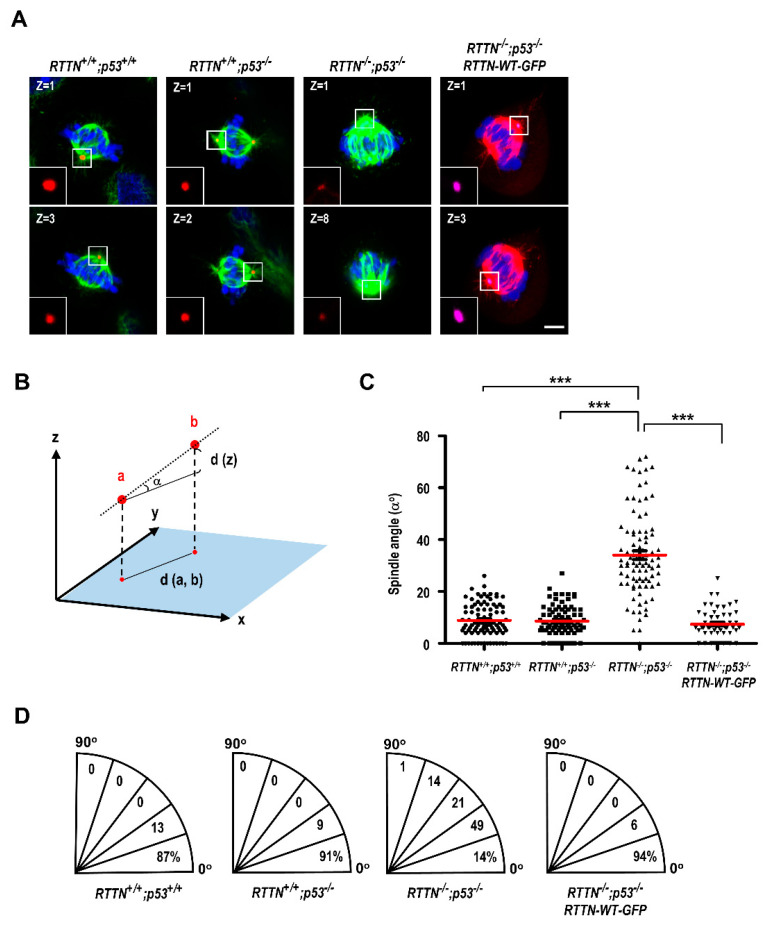
Loss of *RTTN* causes spindle misorientation during mitosis. (**A**) RPE1-based *RTTN^+/+^*; *p53^+/+^*, *RTTN^+/+^*; *p53^−/−^*, or *RTTN^−/−^*; *p53^−/−^* cells were treated as described in Figure 3F. Metaphase cells were then fixed by methanol and immuno-stained with antibodies against CDK5RAP2 (red) and α-tubulin (green) to define the mitotic spindle poles. (**A**, the right-most panel) The spindle misorientation in *RTTN^−/−^*; *p53^−/−^* cells can be effectively rescued by exogenously expressing *RTTN-WT-GFP* construct (CDK5RAP2, purple; α-tubulin, red). Representative images of individual z-stack (0.95 μm per stack) with maximum CDK5RAP2 intensity in metaphase cells are shown. DNA was counterstained with DAPI. (**B**) The schematic diagram illustrating the spindle angle calculation. The xy plane linear distance (d (a, b)) and the z plane vertical distance (d (z)) between two spindle poles (a and b) obtained from a series of z-stack images were used to calculate the spindle angle α. (**C**) Quantification data of the spindle angles in metaphase cells. (**D**) Paradigms illustrating the distribution of spindle angles in metaphase cells (*n* = 90 for *RTTN^+/+^*; *p53^+/+^*, *RTTN^+/+^*; *p53^−/−^*, and *RTTN^−/−^*; *p53^−/−^* groups; *n* = 53 for *RTTN*-WT-GFP). Data are represented as the mean ± SEM from more than three independent experiments. Data are analyzed by one-way ANOVA with Tukey’s multiple comparison test. *** *p* < 0.0001. Scale bars: 5 μm.

Next, we investigated whether loss of *RTTN* could induce spindle misorientation. As shown in Figure 5, *RTTN* loss did produce spindle misorientation in *RTTN^−/−^*; *p53^−/−^* cells, while exogenously expressing *RTTN*-WT-GFP in *RTTN*^−/−^; p53^−/−^ cells can rescue this phenotype. This finding suggests that *RTTN* regulates spindle orientation in mitotic cells.

## 4. Discussion

Recessive mutations in the *RTTN* gene were identified as a cause of primary microcephaly, polymicrogyria, and lissencephaly, which exhibit heterogeneous clinical phenotypes and cerebral malformations. We and others previously showed that *RTTN* (Rotatin) is a centriolar protein that participates in centriole elongation during interphase [11], and *RTTN* mutant cells exhibited severe mitotic failure with centrosome amplification and multipolar spindle formation [22]. However, the molecular mechanism of how *RTTN* functions in mitosis remains largely unknown. Here, we report that complete loss of *RTTN* resulted in PCM dispersion and multiple mitotic abnormalities (e.g., monopolar, abnormal bipolar, and multipolar spindle formation), leading to aneuploidy and apoptosis (Figure 1 and Figure 2), consistent with a previous report [22]. Importantly, *RTTN* loss impeded the congression of NuMA and p150Glued at the mitotic spindle poles, caused the mis-localization of cortical NuMA (Figure 3), and reduced the number and the length of astral microtubules (Figure 4).

Proper cell division relies on accurate mitotic spindle positioning. In mammalian cells, the mitotic spindle positioning is mostly governed by the evolutionary conserved ternary complex NuMA/LGN/Gαi, which serves to anchor the microtubule minus end-directed motor protein complex dynein at the cell cortex [32]. Thus, the cortical dynein positions the spindle by exerting pulling forces on astral microtubules and/or by anchoring the plus-end of astral microtubules to the cortex [27,32,33]. In this study, we found that loss of *RTTN* caused PCM dispersion (Figure 1A), altered NuMA/p150Glued congression to the spindle poles (Figure 3A–C), induced mis-localization of cortical NuMA (Figure 3A,D), and destabilized astral MTs (Figure 4A). Importantly, we found that loss of *RTTN* causes spindle misorientation (Figure 5). Together, our findings suggest that *RTTN* may have a role in maintaining spindle pole integrity and spindle position, at least in part, through a NuMA/dynein-mediated process during mitosis.

Recently, several *MCPH* proteins (e.g., *WDR62*, *CDK5RAP2*, *CENPJ/CPAP*, and *STIL*) were reported to be involved in regulating spindle position, centrosome duplication, centrosome integrity, and/or microtubule stabilization [6,9,14,29,34,35]. Mitotic spindle orientation is essential for cell fate decisions, particularly in neuronal progenitor cells (NPCs) that divide symmetrically or asymmetrically to produce different cell fates during neurogenesis [36,37]. Intriguingly, the loss of *WDR62*, *CDK5RAP2*, and *ASPM* induced the spindle misorientation of NPCs, leading to premature neuronal differentiation in the developing cerebral cortex [34,38,39]. Furthermore, our recent data demonstrated that conditional *Cpap* deletion in the central nervous system preferentially induces multiple mitotic abnormalities with predominantly monopolar spindles in NPCs, perturbs symmetric NPC divisions, and promotes premature neuronal differentiation [40]. Here, we found that loss of *RTTN* induces multiple mitotic abnormalities and perturbs cortical NuMA localization and astral microtubule stabilization. We hypothesize that such an alteration in mitotic NPCs may induce spindle positioning defects, resulting in impeding NPC division mode and promoting premature neuronal differentiation. Future investigation of the shRttn-treated developing brain or conditional *RTTN* knockout mice may elucidate this possibility.

Finally, our flow cytometry data showed that a significant increase in the sub-G1 apoptotic cells (<2 N) and aneuploidy cells (>4 N) was observed in *RTTN^−/−^*; *p53^−/−^* cells (Figure 2D, E). The molecular mechanism is not clear. Interestingly, the depletion of *CPAP* also induced multiple mitotic abnormalities [29], a pattern similar to *RTTN*-knockout cells described here and *RTTN*-mutated fibroblasts described by Vandervore et al. [22]. It is possible that loss of *RTTN* produces structural and functional centriole aberrations, which perturbs the assembly of functional centrosomes (spindle poles) in mitotic cells, leading to multiple mitotic spindle defects, aneuploidy, apoptosis, and spindle misorientation. Furthermore, acentriolar mitosis frequently activates a p53-dependent apoptotic pathway [40,41,42,43,44,45]. Strikingly, a small portion of apoptotic cells (sub-G1, 5.6 ± 1.3%) was observed in *RTTN^−/−^*; *p53^−/−^* cells (Figure 2D) in the absence of p53, implying that a p53-independent apoptosis occurred in *RTTN*-knockout cells. Future studies are needed to resolve its underlying reason. 

## 5. Conclusions

We showed that the human microcephaly protein *RTTN* plays an essential role in mitosis. *RTTN* is required for proper mitotic progression, the maintenance of spindle pole integrity, and correct spindle position. 

## Data Availability

All the data presented in this study are included in this article.

## Supplementary Materials

The following are available online at https://www.mdpi.com/article/10.3390/cells10061441/s1, Figure S1: Uncropped images of Western blots shown in Figure 3E.
