# Supplementary figures and images for: Human Microcephaly Protein RTTN Is Required for Proper Mitotic Progression and Correct Spindle Position
Source: Cells. 2021 Jun 9;10(6):1441. doi: 10.3390/cells10061441 (PMC8229632; doi:10.3390/cells10061441)

**Supplementary Figure 1. Uncropped images of Western blots shown in Figure 3E.**

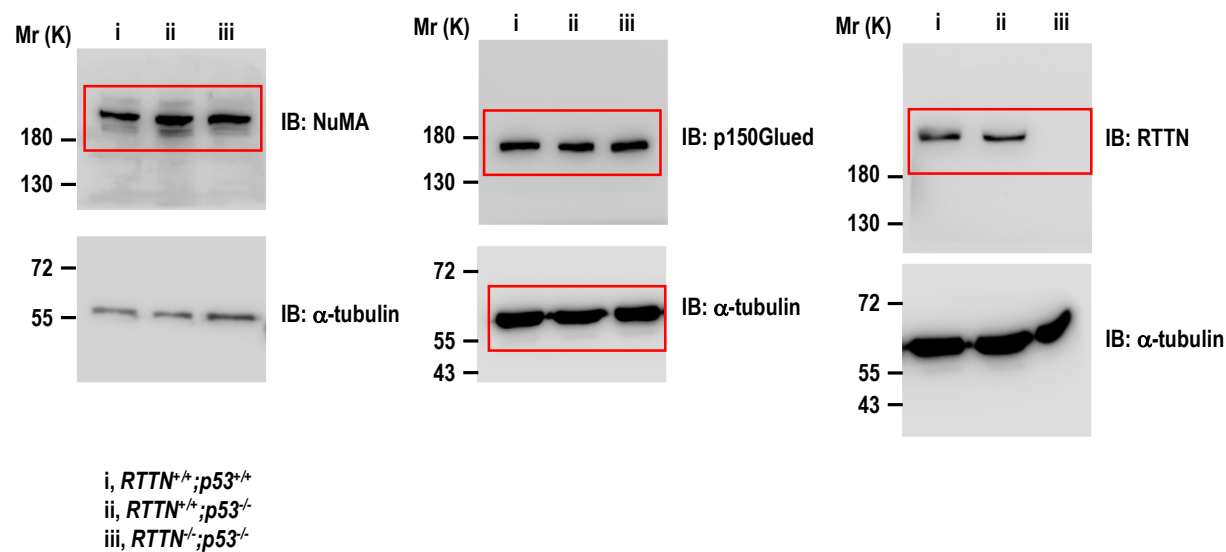

Supplement: Supplementary file 1 [file cells-10-01441-s001.zip › cells-1208323-supplementary.pdf]
